# Healing potential of art therapy: a narrative review of neuro-psycho-cultural mechanisms in mental health

**DOI:** 10.3389/fpsyg.2025.1662772

**Published:** 2026-01-19

**Authors:** Jiarong He, Zhengyi Zhang

**Affiliations:** 1 College of Fine Arts & Design, Tianjin Normal University, Tianjin, China; 2 Department of Dermatology, The First Affiliated Hospital of Xi'an Jiaotong University, Xi'an, Shaanxi, China

**Keywords:** art therapy, mental health, neuroaesthetics, biological foundations, neural mechanisms

## Abstract

**Introduction:**

Art therapy (AT) is a non-pharmacological complementary intervention that integrates biological, psychological, and cultural dimensions of healing through creative expression. This narrative review aims to synthesize the historical evolution, theoretical foundations, and clinical applications of AT, proposing a neuro-psycho-cultural framework to elucidate its therapeutic mechanisms.

**Methods:**

We conducted a purposive literature search in PubMed, Web of Science, and PsycINFO (1990-2025) using keywords such as “art therapy,” “neuroaesthetics,” “default mode network,” and “interpersonal neural synchrony”, etc. Studies were selected based on their relevance to constructing a neuro-psycho-cultural model of AT.

**Results:**

AT facilitates healing through multiple mechanisms: (1) neurobiologically reconfiguring the default mode network (DMN), salience (SEN), and central executive networks (CEN), enhancing interpersonal neural synchrony (INS), and modulating biomarkers; (2) psychologically fostering self-identity, emotion regulation, and flow states; (3) culturally, adaptive frameworks that validate its transcultural applicability. Clinical applications demonstrate AT's benefits in trauma, schizophrenia, neurodegenerative diseases, and cancer-related symptoms.

**Discussion:**

The neuro-psycho-cultural framework positions AT as an integrative, patient-centered intervention that bridges neuroscience, psychology, and cultural anthropology. Despite promising evidence, future research should prioritize rigorous controlled trials, standardized outcome measures, and cross-cultural validation to fully establish AT's efficacy and mechanisms.

## 1 Introduction

### 1.1 The paradox of art: universality and diversity

Visual art represents a fundamental aspect of human behavior, with both cross-cultural prevalence and culture-specific variations in expression. This diversity, rooted in the organization and function of the human brain, aligns with its biological universality rather than eluding scientific inquiry (Nadal and Chatterjee, 2019). From prehistoric cave paintings to contemporary digital art, its capacity to evoke emotion, shape identity, and foster connection transcends time and geography. This duality has sparked interest in its therapeutic potential. While traditional medicine often focuses on symptom reduction, AT offers a complementary approach by addressing the psychological, social, and neural dimensions of mental health (Hu et al., 2021). Through creative processes (e.g., painting, sculpture, and music), AT aims to restore fragmented self-narratives, regulate emotions, and promote resilience (Douglas, 2017).

### 1.2 Defining art therapy

We define art therapy (AT) as a professional psychotherapeutic intervention involving three essential components: (1) a trained art therapist, (2) a client or patient, and (3) active engagement in art-making as the primary mode of communication and therapeutic change (American Art Therapy Association, AATA; British Association of Art Therapists, BAAT). This definition explicitly distinguishes AT from (a) general art-making activities without therapeutic intent, (b) passive art appreciation or aesthetic experiences, and (c) other creative art therapies (e.g., music and dance therapies). However, this distinction does not imply isolation. Insights from each of these three domains can meaningfully inform our understanding of AT's underlying mechanisms, as they share overlapping neurobiological, psychological, and relational processes that converge with AT's therapeutic action.

### 1.3 AT: from ancient rituals to scientific discipline

**Figure 1:**
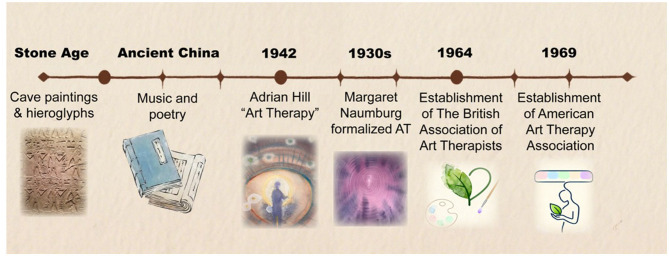
The Brief History of AT. Images adapted with permission from Prof. Hong Li.

The therapeutic use of visual expression dates back to antiquity, with ancient practices, such as ancient Chinese texts and Stone Age cave art, providing historical precedents for modern art therapy's focus on emotional expression and psychological healing (Haslam, 2011; Bogen, 2016) (Figure 1). These early practices facilitated memory, emotional expression, and spiritual sustenance across cultures. Eastern traditions formally acknowledged the therapeutic value of art. For example, China's Qing Dynasty text “*Theoretical Parallel Prose*” (理论骈文) prescribed literature and music to alleviate emotional distress, considering these forms superior to medicinal treatments. The modern concept of AT emerged in the mid-20th century: Adrian Hill coined the term “art therapy” during his 1942 tuberculosis treatment, while Margaret Naumburg formalized the discipline through psychoanalytic integration, culminating in the establishment of the American Art Therapy Association in 1969 (Detre et al., 1983). McNiff (1981) characterized AT as one of the most ancient forms of healing. The British Association of Art Therapists later defined AT as “psychotherapy using art media as its primary communication mode.” In the late 20th century, AT underwent scientific transformation through Semir Zeki's neuroaesthetics, which established biological mechanisms that connect artistic experiences to brain reward systems, self-reflection, and empathy (Zeki, 1999; Kolodny, 2020; Magsamen and Ross, 2024). Contemporary frameworks now integrate neuroscience with anthropology and psychology, validating the efficacy of AT while expanding accessibility through cultural hybridization and social prescribing (Mughal et al., 2022; Magsamen and Ross, 2024).

### 1.4 Scope and synthesis of this narrative review

This narrative review employed a purposive sampling strategy to identify seminal and contemporary literature relevant to our neuro-psycho-cultural framework, aiming to provide a comprehensive and interdisciplinary synthesis of the healing potential of AT. We conducted targeted searches in PubMed, Web of Science, and PsycINFO databases (search period: 1990–2025) using combinations of the following terms: “art therapy,” “neuroaesthetics,” “sense of self,” and related neural mechanisms (e.g., “default mode network,” “interpersonal neural synchrony”). The selection of literature was guided by its relevance in constructing the central thesis of a neuro-psycho-cultural triad in AT, prioritizing impactful empirical studies, foundational theoretical papers, and illustrative clinical cases across populations (trauma, schizophrenia, and neurodegenerative diseases). The following synthesis is structured to explore how AT nurtures the sense of self. It will delineate the supporting neurobiological and psychological mechanisms, examine its clinical applications and cultural dimensions, and conclude with a discussion of integrated findings and future perspectives.

## 2 Synthesis of findings: a neuro-psycho-cultural framework

### 2.1 Interdisciplinary boundaries and evidence integration

In constructing this neuro-psycho-cultural framework, we consciously integrate findings from adjacent fields to enrich our mechanistic explanations. To ensure conceptual clarity, we define the scope of the evidence as follows:

The primary foundation is direct AT evidence. This comprises clinical studies in which visual art-making is the core therapeutic modality within a defined psychotherapeutic relationship. To elucidate specific therapeutic mechanisms, we also draw upon parallel evidence from related creative art therapies, such as music and dance/movement therapies. These studies are cited when they provide robust paradigmatic examples of a therapeutic mechanism that is theoretically transferable to AT. We explicitly note when cited evidence originates from these analogous modalities. Furthermore, we incorporate foundational insights from neuroaesthetics—the study of the brain's response to art in non-clinical settings. Neuroaesthetics, pioneered by Semir Zeki in the late 20th century (Zeki, 1999), investigates the neural mechanisms underlying aesthetic experiences, artistic creation, and appreciation. This interdisciplinary field merges neuroscience, psychology, and art studies to explore how the brain processes beauty, emotion, and meaning in both natural and artistic contexts (Nalbantian, 2008). These studies are cited to bridge basic science with clinical applications. This integrative approach allows us to present a more comprehensive and mechanistic synthesis while maintaining scientific specificity by transparently acknowledging the source and the inferential distance of evidence.

### 2.2 Key findings

Contemporary research has provided preliminary neurobiological evidence supporting AT as a potentially effective therapeutic intervention, although the underlying mechanisms remain to be definitively established. To ensure conceptual clarity, we establish a hierarchical evidence framework:

(1) Primary evidence: Direct art therapy intervention studies with clinical populations.
(2) Supporting evidence: Neuroimaging studies related to artistic creation in non-clinical populations.
(3) Theoretical insights: Findings from related creative art therapies provide paradigmatic examples of transferable mechanisms. Each evidence type will be explicitly labeled to maintain scientific specificity and prevent conceptual conflation.

Thus, the core findings include (a) neurobiological reorganization associated with AT; (b) clinically significant symptom reduction in populations with trauma, schizophrenia, stroke, and cancer; and (c) culturally embedded frameworks that facilitate cross-cultural healing. Table 1 summarizes the key studies supporting these findings, with detailed information on sample sizes, methods, and limitations. Notably, the majority of neuroimaging studies on AT are small-scale proof-of-concept designs, and causal links between specific AT techniques and neural changes remain unconfirmed because of methodological heterogeneity and the absence of large-scale randomized controlled trials.

**Table 1:** Key findings.

**Neurobiological mechanisms**					
**Article title**	**Author (year)**	**Topics and key findings**	**Sample sizes**	**Methods**	**Limitations**
How art changes your brain: differential effects of visual art production and cognitive art evaluation on functional brain connectivity	Bolwerk et al., 2014	Topics: DMN Reconfiguration and AT Key findings: Visual art-making enhances DMN functional connectivity	28 post-retirement adults (63.71 years ± 3.52 SD)	fMRI	Small sample size; lack of clinical population Small sample size; focus on passive appreciation (neuroaesthetics) rather than active AT
The brain on art: intense aesthetic experience activates the default mode network	Vessel et al., 2012	Topics: Improved self-referential processing in AT Key findings: Aesthetic experiences activate DMN, integrating self-referential and emotional processing	11 male (27.6 ± 7.7 years)	fMRI and behavioral analysis	
EEG hyperscanning and qualitative analysis of moments of interest in music therapy for stroke rehabilitation—a feasibility study	Tucek et al., 2022	Topics: Music therapy for stroke rehabilitation Key findings: INS is enhanced during collaborative music therapy sessions	1 stroke patient, 1 therapist	EEG hyperscanning	Single-case design; limited generalizability Single-case design; limited generalizability
Intra- and inter-brain coupling and activity dynamics during improvisational music therapy with a person with dementia: an explorative EEG-hyperscanning single case study	Maidhof et al., 2023.	Topics: Music therapy with a person with dementia Key findings: Within-session differences in neural synchronization and musical features highlight the dynamic nature of music therapy.	1 person with dementia	EEG hyperscanning	
Reduction of cortisol levels and participants' responses following art making	Kaimal et al., 2016	Topics: Cortisol levels and AT Key findings: Art-making reduces cortisol levels	39 healthy adults (18–59 years)	Salivary cortisol measurement	Lack of active control group; short-term follow-up
Increased functional connectivity in military service members presenting a psychological closure and healing theme in art therapy masks	Payano Sosa et al., 2023	Topics: at of mask-making interventions Key findings: at increased functional connectivity, correlated with psychological closure	104 military service members	fMRI	Small sample; no long-term outcome data
Art therapy may reduce psychopathology in schizophrenia by strengthening the patients' sense of self: a qualitative extended case report.	Teglbjaerg, 2011	Topics: Strengthened self-concept through AT externalization Key findings: AT reduces psychopathology by reinforcing self-concept	5 patients with schizophrenia 5 patients with nonpsychotic psychiatric disorders	interviews and written evaluations before and after therapy and at a 1-year follow-up	Small sample; subjective outcome measures
Feasibility of visual art therapy (VAT) on rehabilitation of post-stroke patients.	Zavanone et al., 2024	Topics: assess the feasibility and benefits of VAT Key findings: Marked improvement of cognitive performances through VAT	23 patients (13 women, 10 men, mean age of 70.43 ±12.15)	Evaluation scales	Small sample; absence of a standardized and reproducible score
The effect of art therapy on pain, emesis, anxiety, and quality of life in operated breast cancer patients: randomized control trials	Mollaoğlu et al., 2024	Topics: AT efficacy on operated breast cancer patients Key findings: AT decreased pain, nausea-vomiting, and anxiety levels	60 patients with breast cancer	Evaluation scales	Short follow-up period; intervention specificity
Impact of group art therapy using traditional chinese materials on self-efficacy and social function for individuals diagnosed with schizophrenia	Tong et al., 2021	Topics: Impact of traditional Chinese materials Key findings: AT improved self-efficacy and social function, reducing social and life function problems	104 patients with schizophrenia	Evaluation scales	Linguistic issues; only traditional Chinese materials
Creative arts, culture, and healing: building an evidence base.	Archibald and Dewar, 2010	Topics: how creative arts are used in Indigenous healing programs across Canada Key findings: Creative arts, culture, and healing are linked to each other	104 healing programs	Questionnaire	Lack of standardized evaluation tools; lack of control groups

### 2.3 AT nurturing the self

**Figure 2:**
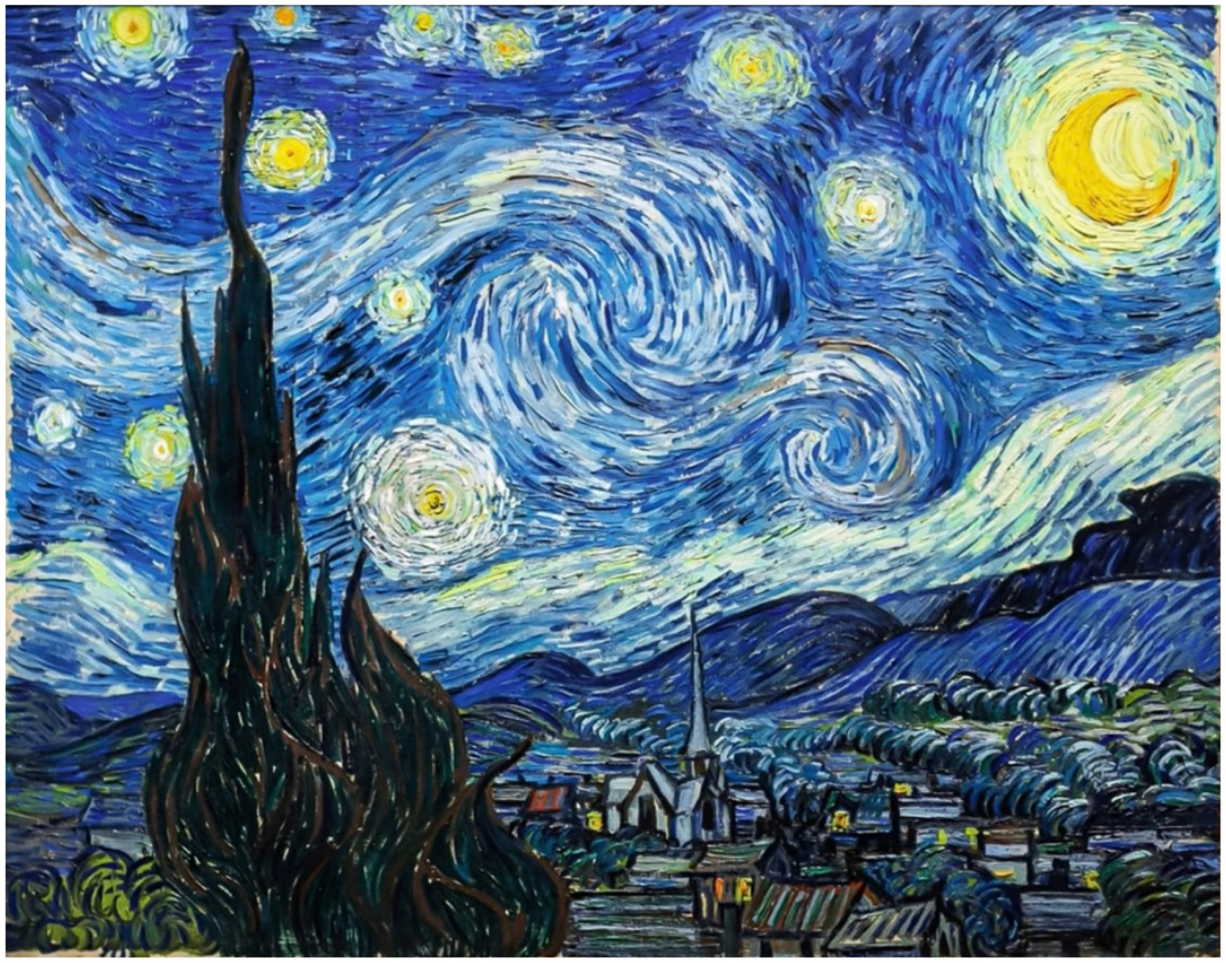
The Starry Night, Vincent Van Gogh, 1889. (In the public domain, sourced from: https://www.nbfox.com/vip-only-popular-artists-paintings/).

A sense of self or personal identity can be defined by a unique set of psychological, interpersonal, and physical attributes constituting an individual's identity, which also serves as the foundation for daily experiences (Moe and Docherty, 2014). Disturbances in the sense of self are central to schizophrenia, depersonalization disorders, anosognosia, Capgras syndrome, bipolar disorder, and other disorders (Inder et al., 2008; Johnstone et al., 2020; Kirberg and Chadha, 2024). Creative freedom in AT allows patients to externalize and process emotions and experiences that may be ineffable through verbal means, fostering self-sufficiency and integrating the self into therapy (de Witte et al., 2021). The concept that art can serve psychological functions has historical roots. Philosophers such as Aristotle theorized about art's cathartic potential. Similarly, personal accounts of artists, such as Vincent Van Gogh, illustrate the relationship between creative expression and psychological states (Figure 2: *The Starry Night*). Although the personal significance of art has been illustrated, the practice of modern AT to facilitate psychological transformation through symbolic representation and emotional processing requires proper facilitation.

While long-term pharmacotherapy ameliorates the positive symptoms of psychosis, residual negative symptoms persist, affecting behavioral repertoires. Adjunctive AT promotes holistic identity development and enhances social communication (Hanson et al., 2010). Studies confirm that AT reduces schizophrenia psychopathology by reinforcing self-concept (Teglbjaerg, 2011), providing a safe space for emotional embodiment and cognitive integration (Samaritter, 2018; Vaisvaser, 2021; Vaisvaser et al., 2024). Notably, marginalized groups (e.g., individuals with learning disabilities) use art to reclaim agency and reshape societal perceptions (Hall, 2013). Similarly, military personnel with post-traumatic stress disorder (PTSD) and traumatic brain injury benefit from the trauma-processing capacity of AT, which helps alleviate guilt and restores self-continuity (Kaimal et al., 2019). Furthermore, evidence from structured art-making contexts, including some therapeutic art programs, suggests potential benefits for empathy and self-esteem in various populations (Won, 2016). Separately, research on arts education has linked artistic engagement to the growth of critical thinking and self-concept (Hinds, 2017). While these findings from adjacent fields are suggestive, the highlighted mechanism may be operationalized systematically within a formal AT context. These outcomes align with AT's role in facilitating externalization-concretization and symbolization, which are core mechanisms for affective-cognitive processing (de Witte et al., 2021; Vaisvaser et al., 2024). In summary, the synthesized literature suggests that the cultivation of the sense of self is a central proposed mechanism through which AT may address disturbances in self-experience across different mental health conditions.

### 2.4 Integrative framework of AT: neuro-psychological-cultural triad in art healing

#### 2.4.1 Neurobiological mechanisms of healing: an integrated network analysis

Synthesis of neuroimaging literature suggests that AT's therapeutic effects may be mediated by the coordinated reconfiguration of several large-scale brain networks. This analysis moves beyond theoretical models to focus on empirical findings linking art making to measurable neural changes. Although causal relationships remain to be definitively established through longitudinal studies, the evidence points to a core mechanism: AT facilitates a shift from maladaptive, often rigid, neural states toward a more integrated and flexible functional architecture, primarily through the modulation of the Mirror Neuron System (MNS), the Default Mode Network (DMN), the Salience Network (SEN), the Central Executive Network (CEN), and the promotion of Interpersonal Neural Synchrony (INS). The following synthesis delineates how these changes underpin key therapeutic processes, such as self-referential processing, emotion regulation, and social connection.

##### 2.4.1.1. Mirror neuron system (MNS) and AT

Viewing visual artworks can activate the motor cortex and MNS, which refers to the interaction between the superior temporal sulcus (STS), posterior parietal cortices (PPC), and Broca's area (Sariñana, 2024). For instance, functional magnetic resonance imaging (fMRI) studies reveal that viewing dynamic human actions in artworks (e.g., dance, sculptures) activates the premotor (vPMC) and PPC, reflecting embodied simulation of implied movements (Di Dio et al., 2007; di Dio et al., 2016). Similarly, transcranial magnetic stimulation (TMS) studies demonstrate increased corticospinal excitability (CSE) during the observation of dynamic human-action paintings, correlating with perceived dynamism and aesthetic preference (Fiori et al., 2020). It can be logically assumed that these acts and all others involved in creating a piece of art on an embodied simulation level could activate the artist's MNS in Broca's area, enhancing the possibility for later verbalization. Thus, art engagement may enhance motor resonance and empathy through MNS activation, supporting non-verbal emotional processing in therapy.

##### 2.4.1.2. The dynamic interplay of DMN, SEN, and CEN in AT

A critical finding across studies is that AT can modulate the interplay between the DMN, SEN, and CEN. The DMN, central to self-referential thought and autobiographical memory, often shows dysregulation in conditions such as depression and PTSD (Lanius et al., 2015; Kearney and Lanius, 2022). Empirical evidence from AT contexts indicates that art-making can recalibrate DMN activity. For instance, Bolwerk et al. directly demonstrated that visual art-making specifically enhanced DMN functional connectivity (Bolwerk et al., 2014), particularly between the PCC/precuneus and frontal/parietal regions, suggesting a neural correlate of improved self-integration. Moreover, the hyperactivity of DMN in disorders is modulated through art-induced embodied metaphor and somatic-cognitive integration, with fMRI studies confirming altered DMN connectivity following AT (Payano Sosa et al., 2023). While passive aesthetic experiences have been shown to activate the DMN, integrating self-referential and emotional processing (Vessel et al., 2013; Bolwerk et al., 2014; Chatterjee and Vartanian, 2014; Belfi et al., 2019), it remains unclear whether active art-making within therapeutic contexts produces similar neural patterns. Future studies should directly compare these conditions. Collectively, art-induced reward system activation (e.g., dopamine pathways) may alleviate depressive symptoms and reinforce positive affect.

**Figure 3:**
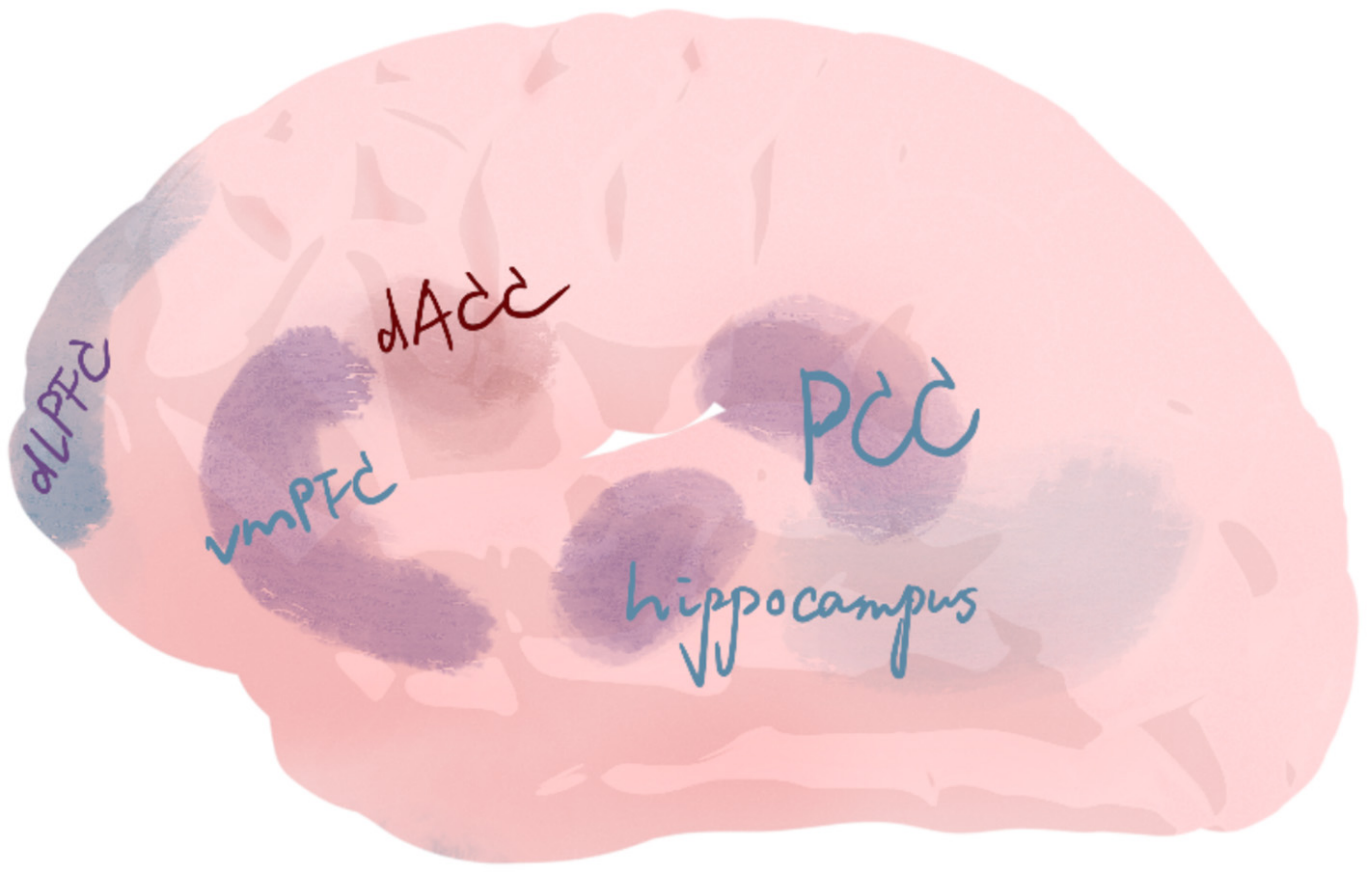
Brain networks involved in AT.

The interplay is facilitated by the SEN (linked to attention and emotion regulation), which acts as a “switch” between the DMN and CEN (linked to working memory and cognitive control) in AT (Lusebrink and Hinz, 2020; van Leeuwen et al., 2022). The inherently salient nature of creative expression is hypothesized to engage the SEN, directing attention to internally generated stimuli (emotions, memories) and then recruiting the CEN for cognitive reappraisal and executive control over the artistic medium. This proposed SEN-mediated cycle—from somatic and emotional awareness (supported by INS, discussed below) to cognitive reframing—provides a testable model for understanding how AT helps in integrating fragmented self-experiences, a mechanism corroborated by clinical observations in Parkinson's patients post-AT (Cucca et al., 2021), mild cognitive impairment patients after AT (Yu et al., 2021), and during creative cognition (Beaty et al., 2017) and art-making (Ellamil et al., 2012; De Pisapia et al., 2016). These interdependent network reconfigurations underscore the need to study the healing mechanisms of AT through an integrated neural system lens (Figure 3). However, it should be noted that the direct measurement of SEN and CEN engagement during AT sessions remains an area for future research, with current models often inferring their roles from broader neurocognitive frameworks.

##### 2.4.1.3. Extending the framework: interpersonal neural synchrony (INS) in AT

The network dynamics described above are profoundly influenced by the interpersonal context of therapy, with INS serving as a core neurobiological mechanism, which is defined as brain-to-brain coupling between individuals during interactive experiences (Vaisvaser et al., 2024). Scanning studies, although more prevalent in music therapy, provide a crucial mechanistic analog for AT. While direct evidence from art therapy remains limited, parallel evidence from related creative therapy fields shows that joint artistic engagement reliably induces INS, such as while or after creating, performing or listening to music (Abrams et al., 2013; Sachs et al., 2020; Liu et al., 2021; Khalil et al., 2022; Ramírez-Moreno et al., 2022), performing dramatic scenes (Greaves et al., 2022), viewing a dance performance (Herbec et al., 2015) or a movie (Dziura et al., 2021), and through collaborative drawing (Gonçalves da Cruz Monteiro et al., 2022) and coordination movement (Basso et al., 2020; Varlet et al., 2020). This neural coupling is considered to underpin the development of a therapeutic alliance, shared understanding, and empathy. We posit that INS serves as a foundational interpersonal substrate that facilitates intrapersonal network changes, as previously described. For example, the safety and connection established through INS may allow a client to engage more freely with distressing self-referential material in the DMN, whereas a shared focus (a form of external SEN activation) supports co-regulation. Preliminary evidence from electroencephalography (EEG) in music therapy with clinical populations (Tucek et al., 2022; Kang et al., 2023; Maidhof et al., 2023) supports the feasibility and clinical relevance of this approach. Translating these paradigms into visual AT settings is a critical next step in the field. To date, the relational aesthetic engagement in AT could help cultivate spontaneous INS during the interaction (Vaisvaser et al., 2024). With the development of neuroscience and neuroaesthetics, we can gradually understand how arts and aesthetic experiences physically change our brains, bodies, and behaviors, and how we can better utilize AT in health, learning, and community development (Galiatsatos et al., 2024).

In summary, the neurobiological framework for AT is evolving from a collection of intriguing correlations to a testable model of integrated brain network changes. The synthesized evidence suggests that AT does not engage a single “art center” but rather facilitates healing through the coordinated recalibration of multiple systems: the DMN for self-integration, the SEN for affective salience, the CEN for cognitive control, and INS for relational safety. This multi-level mechanism aligns with the neuro-psycho-cultural framework, providing a biological basis for how artistic creation can simultaneously rebuild the self within an individual and connect them to others. Future studies should prioritize causal longitudinal designs and standardized neuroimaging protocols to move from this synthesized model to a more definitive evidence.

#### 2.4.2 Psychological foundations: positive psychology and flow

AT is deeply rooted in psychological principles, particularly those of positive psychology, which emphasize strength, wellbeing, and human flourishing (Staricoff, 2006; Stuckey and Nobel, 2010). First, AT fosters positive emotions such as joy, hope, and gratitude through creative expression (Lanius et al., 2011). Studies within art therapy contexts demonstrate that structured art-making with therapeutic intent, when facilitated by trained professionals, can significantly improve mood and reduce stress in clinical populations (Curl, 2008; Henderson et al., 2009). Second, flow and engagement are other ways to shape the psychology of happiness (Csikszentmihalyi, 2013). The immersive nature of art-making induces flow, a state of focused engagement in which time perception dissolves and skills align with challenges. Flow states have been associated with improved mastery and optimism in some therapeutic contexts (Seligman et al., 2006), although causal relationships specific to art therapy require further empirical validation through controlled studies. Moreover, AT interventions, such as open-studio practices, explicitly cultivate flow, which correlates with emotion regulation and post-traumatic growth (Wilkinson and Chilton, 2013; Chilton and Wilkinson, 2016). Third, AT helps in meaning-making and post-traumatic growth, and it facilitates the construction of meaning through symbolic expression, helping individuals to reframe trauma into narratives of resilience (Tedeschi and Calhoun, 2004). For instance, cancer survivors use art to visualize “altars of resilience,” transforming adversity into purpose (Collie et al., 2006). This aligns with positive psychology's focus on benefit-finding and identifying strengths forged through struggles (Linley and Joseph, 2004). In short, an integrative psychotherapy approach that uses AT can identify the emotional state of a patient and serve as a useful aid to help stroke patients in the rehabilitation process (Eum and Yim, 2015). Finally, AT assessments and interventions, such as visualizing “signature strengths” or creating family trees of resilience, operationalize positive psychology's taxonomy of virtues (e.g., creativity, curiosity) (Peterson and Seligman, 2004). Research on male art therapists revealed that appreciation of beauty and curiosity were dominant strengths, underscoring art's role in self-actualization (Riddle and Riddle, 2007). The integration of positive psychology principles with AT's non-verbal expressive capacities provides a theoretical basis for addressing symptom reduction and the promotion of adaptive psychological functioning, including resilience and meaning-making (Wilkinson and Chilton, 2013). The empirical validation of these combined effects in art therapy remains an area for future research.

#### 2.4.3 Anthropology and culture foundations

The therapeutic potential of art is deeply intertwined with cultural and anthropological frameworks, bridging indigenous practices, community engagement, and cross-cultural sensitivity. Anthropological and historical records indicate that artistic expressions, such as the creation of grave decorations, memorial textiles, and altars, have served psychological and social functions in the processes of grieving, loss, and cultural meaning-making across diverse populations (Westrich, 1994). These cultural precedents highlight the ability of artistic expression to mediate psychological experiences, a capability that AT systematically harnesses within a structured psychotherapeutic framework. Indeed, it is essential to recognize the need for multicultural competence and diversity, along with sensitivity to one's own and clients' cultural awareness and cultural heritage, including how aesthetic experience and artistic experience all influence the process of AT (Arslanbek et al., 2022). These practices align with anthropological methods that prioritize participant-driven narratives and cultural meaning-making (McCurdy et al., 2004). For example, traditional art forms such as weaving and embroidery help clients reconnect with cultural roots disrupted by colonialism and intergenerational trauma, such as residential schools (Archibald and Dewar, 2010; Whyte, 2018). AT practices further resonate with the art course's focus on collaborative art projects with seniors, whereas cyanotype prints and textile exhibitions became media for cultural reconnection (Hoag, 2024). AT's commitment to understanding the client's subjective, embodied experience shares a conceptual affinity with anthropological methods such as ethnographic interviews, which prioritize a deep, contextual understanding of human experience (Vivanco, 2016). This parallel suggests that anthropological sensibilities can inform a culturally responsive therapeutic stance, whereas the direct application of specific anthropological methods within AT requires further research. Studies of community-based art practices, including rhythmic practices such as needlework or communal art-making, report associations with states of mindfulness and emotional grounding (Collier, 2011; Garlock, 2016). In addition, group art activities (e.g., quilting circles) strengthen social bonds and intergenerational knowledge transfer (Anwar McHenry, 2011; Lu and Yuen, 2012). Researchers have pointed out that pop culture can be integrated into AT for individuals with autism (Stallings, 2022). Moreover, AT can address historical trauma by revitalizing cultural pride and identity (Duran et al., 1998; Edwards, 2020), along with the power of psychosocial intervention to deal with trauma after the Japanese tsunami (Gavron, 2025) or the Chinese earthquake (Han, 2011). Similarly, community-based AT programs blending anthropology and local art forms (e.g., redlining and gentrification) (Hoag, 2024) demonstrate that culturally adaptive frameworks modulate the neuropsychological mechanisms of AT, such as traditional and local art forms (Gómez Carlier and Salom, 2012; Potash et al., 2017; Bonz et al., 2019). However, longitudinal studies are needed to quantify the psychosocial benefits of traditional art interventions and establish ethical frameworks for cross-cultural practices. In conclusion, these findings illustrate that AT can be conceptualized as a discipline that operationalizes the psychological potential of art-making—observed across cultures and history—within modern, ethical, and reflexive practices. It integrates awareness of cultural dimensions (akin to anthropological reflexivity) into the therapeutic process, aiming to facilitate communication and healing that is responsive to the client's cultural background.

### 2.5 Biomarkers in AT: physiological indicator

Historically, art has been studied primarily within the humanities but has not been seriously considered in the sciences (Bromberger et al., 2011). A growing number of scientific studies on neuroaesthetics have elucidated the neural mechanisms underlying art perception and evaluation. With its detected biological foundations, AT has been proposed as a treatment for post-traumatic conditions (Gerge et al., 2025). AT enhances self-understanding, agency, and the capacity to overcome challenges (King, 2016). Neuroscience can be used to support scientific research and the theoretical foundation of AT. The aesthetic engagement in art might induce plasticity of body and peripersonal space representations, especially in an interpersonal context (Drummond, 2024). Recent studies are beginning to quantify the associations between art-making and visual representation with measures of stress, anxiety, and depression (Kaimal et al., 2016; Walker et al., 2017; Kaimal et al., 2018; Berberian et al., 2019). Current research in AT has examined neurobiological changes, including salivary markers of immune function (Futterman Collier et al., 2016), heart rate variability (Haiblum-Itskovitch et al., 2018), EEG (Belkofer et al., 2014; Kruk et al., 2014), fMRI (Walker et al., 2018; Payano Sosa et al., 2023), functional near infrared spectroscopy (fNIRS) (Kaimal et al., 2017), and cortisol levels (Kaimal et al., 2016), which is a key glucocorticoid hormone and widely studied stress marker. Preliminary evidence has suggested an association between AT and reductions in stress and anxiety biomarkers, which is potentially mediated through mechanisms such as cognitive distraction and emotional expression (Reynolds and Prior, 2003; Reynolds and Lim, 2007; Stuckey and Nobel, 2010). However, the causal pathways and specificity of art therapy require further investigation. In addition, blood pressure could also be recognized as a biomarker since it was significantly decreased after art-making, which served as an effective stress reliever. Furthermore, Futterman et al. found that textile art has mood-enhancing effects, as indicated by altered levels of IL-β, a pro-inflammatory cytokine, which reflects changes in inflammatory immune responses (Futterman Collier et al., 2016). Neuroimaging studies show physiological changes during art-making. A preliminary qEEG study found that both clay sculpting and drawing increased gamma power in the right medial parietal lobe compared to general movement (Kruk et al., 2014). Another study also differences in the left posterior temporal, parietal, and occipital regions of artists when comparing pre- and post-drawing EEG recordings within the alpha frequency band (Belkofer et al., 2014). The use of AT for promoting emotional expression, wellbeing, and resilience is well-documented, as art-making can be a basis to consider experiences differently, reorganize thoughts, gain personal insights, and enhance therapeutic relationships (Konopka, 2014; Tavormina et al., 2014; Vaisvaser et al., 2024). These findings contribute to an emerging body of research exploring the biological foundations of AT. Although promising, the current evidence is characterized by methodological limitations, including small sample sizes and inaccurate measurements.

### 2.6 Clinical applications and emerging frontiers of AT

Although AT has been used clinically for more than a century and has been recognized as a profession since 1991, the majority of published works remain theoretical, with limited discussion regarding specific mechanistic outcomes (Devlin, 2006; Junge, 2010). In recent years, systematic and controlled studies have examined the therapeutic effects and benefits of AT (Frisch et al., 2006). For instance, randomized trials in breast cancer patients showed significant reductions in pain, emesis, and anxiety, along with improved quality of life, following marbling art interventions with music accompaniment (Mollaoğlu et al., 2024). Research examining potential biomarkers represents an initial step toward addressing the methodological challenges in AT outcome assessment. Advanced neuroimaging tools (fMRI, fNIRS) and interdisciplinary collaboration are critical for linking biological changes with psychological outcomes (Beans, 2019). Art therapists' clinical expertise could generate testable biopsychosocial hypotheses; however, further research is needed to clarify the unique mechanisms of AT, including (a) therapist–client dynamics: INS during joint art-making, which enhances coordination and relational safety in clinical settings; (b) art effects: differential neural activation patterns; and (c) session structure/duration: optimal duration/frequency, as evidenced by studies showing sustained anxiety reduction in children with asthma after seven weekly sessions (Beans, 2019). Comparative studies should distinguish art therapist-led interventions from artist-led programs (e.g., teaching artists), exploring contextual mediators such as biophilic environments (nature-integrated spaces that lower cortisol) (Miola et al., 2025), traditional studios, and virtual reality spaces (Neill et al., 2019). Theoretical models that propose individualized, neuroscience-informed approaches in AT are conceptually consistent with personalized mental health, as well as culturally adaptive frameworks such as China's Transforming Symptom's Symbol into Emptiness (TSSE), which integrates Qigong and embodied metaphors to resolve psychosomatic symptoms (Niu et al., 2025).

## 3 Discussion

### 3.1 Limitations and future directions

This narrative review has several limitations inherent to its design. The qualitative synthesis and selective inclusion of literature, while suitable for building a theoretical framework, preclude quantitative conclusions and may be subject to selection bias. The field of AT also faces methodological challenges: The majority of included studies have small sizes *n* < 50) and are dominated by Western samples, leading to potential ethnic and cultural bias; positive outcomes are overrepresented in the published literature, suggesting possible publication bias; and few clinical trials use blinded assessors, increasing the risk of detection bias. These biases may overestimate AT's efficacy and limit the generalizability of neurobiological findings (e.g., DMN reconfiguration), highlighting the need for future studies to adopt rigorous designs (e.g., multicenter trials and mixed-methods quality assessment) to mitigate these limitations. Furthermore, standardizing outcome measures and distinguishing AT-specific mechanisms from contextual (e.g., placebo) effects (Abbing et al., 2018) is an important question. Thus, future studies need to prioritize the following: First, employing active control conditions (e.g., structured social activities) to isolate the specific effects of AT from non-specific factors such as therapeutic attention and expectation. Second, establishing a core outcome set that integrates subjective self-report measures (e.g., self-concept scales), objective physiological biomarkers (e.g., cortisol and HRV), and standardized neuroimaging metrics (e.g., DMN connectivity and INS) to facilitate cross-study comparisons. Third, investigating the “active ingredients” and optimal “dosing” of AT, including session frequency, duration, and the differential effects of various art media.

### 3.2 Conceptual and clinical implications

Despite these challenges, the proposed neuro-psycho-cultural framework offers a valuable heuristic model. This framework attempts to integrate subjective healing experiences with objective biological measurements in a cultural context. AT shows promise across diverse populations, with emerging evidence from controlled studies suggesting potential clinical benefits, although larger-scale randomized trials are needed to establish its efficacy. Randomized controlled trials (RCTs) reveal that AT significantly reduces depression in post-stroke patients (Yaqing Yuan and Chen, 2024), alleviates pain, emesis, and anxiety in breast cancer survivors (Mollaoğlu et al., 2024), and enhances spiritual wellbeing and social connectivity in elderly populations with mild-to-moderate depression. Concurrently, interdisciplinary studies, particularly neuroaesthetic investigations of DMN activation, INS synchrony, and stress physiology, further validate the biological and cultural dimensions of AT. For clinicians, this neuro-psycho-cultural framework provides a rationale for using AT to target core self-disturbances in conditions such as PTSD and schizophrenia and justifies the use of culturally adapted practices to enhance engagement and efficacy.

Ultimately, AT represents an integrative intervention paradigm in which creative expression modulates emotional responses, reconstructs self-identity, and enhances communal bonds. This has been increasingly elucidated through scientific research (Banerjee and Roy, 2024). As Semir Zeki posited, the therapeutic value of art is rooted in its congruence with the brain's inherent functional organization. Continued interdisciplinary collaboration will not only validate the clinical efficacy of AT but also clarify the fundamental role of artistic engagement in regulating health, cognition, and interpersonal relationships. With continued development, the gap between the sciences and the humanities has diminished.

## 4 Conclusion

This narrative review synthesized historical, neurobiological, psychological, and cultural evidence to construct a neuro-psycho-cultural framework for art therapy. We conclude that AT is a valid, multi-mechanism intervention that primarily operates by nurturing the sense of self, with supporting evidence from brain networks, physiological biomarkers, and positive psychology. The path forward demands greater methodological rigor, standardized measurement, and a continued commitment to interdisciplinary collaboration to fully validate and refine this model, ultimately optimizing the role of AT in personalized, evidence-based mental health care.

## Data Availability

The original contributions presented in the study are included in the article/supplementary material, further inquiries can be directed to the corresponding author.
